# Laboratory Report of HIV-1 Low-Level Viremia

**DOI:** 10.1093/ofid/ofad667

**Published:** 2024-01-11

**Authors:** Hortensia Álvarez, Josep M Llibre

**Affiliations:** Infectious Diseases Unit, Department of Internal Medicine, Complexo Hospitalario Universitario de Ferrol, SERGAS-A Coruña, Spain; Department of Biochemistry, Genetics and Immunology, Universidade de Vigo, Vigo, Spain; Infectious Diseases Division and Fight Infections Foundation, University Hospital Germans Trias i Pujol, Badalona, Barcelona, Spain

To the Editor—We read with great interest the perspective regarding reporting overly sensitive HIV viral loads by Rodriguez et al. [1]. We respectfully disagree with some statements concerning the absence of implications of HIV viral loads between the lower limit of detection (LLD) and 200 copies/mL, suggesting reporting these levels as “undetectable.” We would like to provide some remarks and clarifications.

The authors assert that there are no clear data indicating that a viral load between the LLD and 200 copies/mL is clinically relevant in portending negative outcomes.

Despite some controversial findings [Table 1, 2–6], there are data from recent cohorts showing an association between low-level viremias (LLVs) with HIV-1 RNA 50–199 copies/mL and clinical outcomes. In a Swedish Cohort, LLV was associated with higher risk of all-cause mortality compared with virological suppression (VS; adjusted hazard ratio [aHR], 2.20; 95% CI, 1.30–3.80). In addition, in a subanalysis, the association with mortality was restricted to participants with ≥25% of HIV-1 RNA measurements ≥50 copies/mL (aHR, 3.30; 95% CI, 1.80–6.40) [4]. In a Chinese Cohort, the incidence of non-AIDS events (including non-AIDS-defining malignancies, cardiovascular, renal, liver, psychiatric, bone, and metabolic diseases) in the LLV stratum was 4.05 (95% CI, 2.06–7.77) per 100 person-years. Indeed, LLV was associated with higher risk of non-AIDS events compared with no LLV (aHR, 4.55; 95% CI, 1.80–11.53) [6]. There are also data supporting this association between LLV and non-AIDS events throughout surrogate biomarkers of cardiovascular damage. HIV-1 RNA 50–199 copies/mL was associated with higher levels of growth differentiation factor 15 (GDF-15) compared with VS. This biomarker is upregulated in response to heart failure and acute coronary syndrome and also in neoplasms such as colorectal and pancreatic cancer [7]. Along the same line, HIV-1 RNA 50–200 copies/mL showed higher levels of intercellular adhesion molecule 1 (ICAM-1), which is an early biomarker of atherosclerosis, compared with VS (adjusted arithmetic median rate, 1.26; 95% CI, 1.07–1.48), in a recent study [8].

**Table 1.** Impact of LLV on Clinical Events Over Time in Cohorts Including Treatment-Naïve Persons With HIV

Reference	Year	Study Design	Population, No.	Definition of LLV	Impact of LLV on AIDS Events	Impact of LLV on All-Cause Mortality	Impact of LLV on Non-AIDS Events
[2]	2015	ART-Cohort Collaboration	17 902	HIV-1 RNA 50–199 c/mL	No^a^ (aHR, 1.11; 95% CI, 0.79–1.61)	No^a^ (aHR, 1.19; 95% CI, 0.78–1.82)	NA
[3]	2018	Spanish CoRIS (RIS cohort)	5986	HIV-1 RNA 50–199 c/mL	Categories AIDS events and mortality, analyzed together: No^a^ (aHR, 1.44; 95% CI, 0.69–3.03)		No^a^ (aHR, 0.81; 95% CI, 0.37–1.75)
[4]	2021	Swedish Cohort	6956	HIV-1 RNA 50–199 c/mL	NA	Yes^b^ (aHR, 2.20; 95% CI, 1.30–3.80)	No^b^ (aHR, 0.86; 95% CI, 0.5–1.5)
[5]	2022	Swedish Cohort	6562	HIV-1 RNA 50–199 c/mL	NA	NA	Cardiovascular disease (ischemic heart disease, stroke, heart failure) No^b^ (aHR, 0.95; 95% CI, 0.45–2.01)
[6]	2022	Chinese Cohort	1288	HIV-1 RNA 50–199 c/mL	NA	No	Non-AIDS-defining malignancies, cardiovascular, renal, liver, psychiatric, bone and metabolic diseases Yes^a^ (aHR, 4.55; 95% CI, 1.80–11.53)

Abbreviations: aHR, adjusted hazard ratio; ART, antiretroviral treatment; LLV, low-level viremia; NA, not applicable.

^a^Compared with no LLV.

^b^Compared with virological suppression.

Likewise, the authors state that viral loads deemed detectable but <200 copies/mL have not demonstrated meaningful, clinically useful or actionable implications.

In this regard, the US Department of Health and Human Services guidelines recommend a thorough assessment of persons with HIV (PWH) and HIV-1 RNA in the range between the LLD and <200 copies/mL. Physicians must specifically assess antiretroviral treatment (ART) suboptimal pharmacokinetics (ie, absorption and metabolism), potency or low barrier to resistance, and adherence and potential drug–drug and drug–food interactions. HIV-1 RNA levels should be monitored at least every 3 months to assess the need for ART genotyping and ART changes in the future. In addition, all guidelines recommend changing the ART to one with a high resistance barrier, namely 3-drug regimens with dolutegravir, bictegravir, or boosted darunavir to avoid emergence of HIV resistance mutations [9].

Of note, data retrieved from a recent study have identified initial baseline high HIV-1 RNA and low CD4+ counts as predictors of subsequent LLV and viral blips. This is probably associated with a greater HIV reservoir and persistent immune damage, and this happens regardless of the type of ART started, including second-generation integrase strand transfer inhibitors or specifically dolutegravir [10].

Taken together, given the above-mentioned statements and data known to date, we think that LLV defined as HIV-1 RNA between the LLD and 200 copies/mL should be considered a different scenario (“detectable” viral load) from HIV-1 RNA below the LLD (“undetectable” viral load) and should be systematically reported. Actually, there are data suggesting increased rates of clinical events and concordant evidence of an increased risk of subsequent virological failure, and treating physicians must undertake several actions. Hence, it seems that LLV has clinical and actionable consequences, in contrast to “undetectable” viral loads.
